# Elevated pulse pressure correlated with reduced retinal peripapillary capillary in thyroid-associated ophthalmology with visual field defect

**DOI:** 10.3389/fendo.2022.941051

**Published:** 2022-09-16

**Authors:** Jie Ye, Weijie Liu, Xiaozhou Hu, Hongxiao Jiang, Mingna Xu, Haochen Jin, Mengting Wang, Zihui Liu, Qi Chen, Wencan Wu, Yunhai Tu

**Affiliations:** School of Ophthalmology and Optometry and Eye Hospital, Wenzhou Medical University, Wenzhou, China

**Keywords:** thyroid-associated ophthalmology (TAO), visual field (VF), optical coherence tomography angiography (OCTA), pulse pressure (PP), retinal capillary

## Abstract

**Purpose:**

To quantify the retinal vessel density in thyroid-associated ophthalmology (TAO) patients with visual field (VF) defect and examine its associations with mechanical and system vascular risk factors for underlying pathogenesis of VF defect in TAO.

**Methods:**

The cohort was composed of 62 TAO eyes (39 with VF defect and 23 without VF defect). The pulse pressure (PP), intraocular pressure (IOP), ophthalmic rectus muscular index (MI), superficial retinal capillary plexus (SRCP), radial peripapillary capillary (RPC) density, and other related parameters were measured. The associations among these factors and VF mean deviation (MD) were analyzed.

**Results:**

In TAO patients with VF defect, reduced RPC density, higher PP, and larger horizontal and vertical MI were found (all P < 0.03) when compared to TAO patients without VF defect. The RPC density was correlated with VF MD value (r = 0.242, P = 0.029), while SRCP density was not (P = 0.419). In univariable general estimating equation (GEE) analysis with RPC density as the outcome, PP and its fluctuation showed a significant association (both P < 0.04). In the final RPC model with multivariable GEE analysis, only PP (β = -0.082, P = 0.029) showed significance while PP fluctuation (P = 0.080) did not.

**Conclusions:**

The elevated PP was correlated with reduced retinal peripapillary perfusion in TAO resulting in VF defect. These data suggested that the system vascular factor may be important in the pathogenesis of reduced retinal perfusion resulting in visual impairment in TAO.

## Introduction

Visual field (VF) defect is one of the most frequent and almost the first clue of vision threats during the progression of thyroid-associated ophthalmology (TAO) (1). The pathogenesis of visual defect in TAO was still unclear. The mechanic compression due to the enlarged rectus muscles and increased orbital volume was widely accepted as the main cause (1). However, despite successful orbital decompression being done, some TAO patients still complained about progressively decreased visual function (2). In Dickinson’s study, even rarely optic nerve stretch was found from the orbital coronal computed tomography scanning images in some TAO patients with extreme proptosis (3). There might be other mechanisms that play the important roles in visual field defect in TAO patients.

The health of the retinal perfusion might be of great importance in maintaining normal visual function. The impaired ophthalmic drainage and related retinal perfusion in TAO with visual impairment had been reported (4, 5). However, the mechanism of retinal perfusion alteration and its association with VF defect in TAO were still unclear. It was well known that abnormal blood pressure contributed to the perfusion damage of the end-organ (6, 7). Our previous study had already found abnormal blood pressure in TAO patients with severe VF defect, although the association between blood pressure and retinal perfusion in TAO with VF defect was unclarified (8). In addition, with continuous mechanic compression, there was a sustained stretching of the optic nerve that might also result in the vascular insufficiency of retinal perfusion (4, 9).

In the current study, we explore the retinal perfusion alteration, as an alternative important mechanism in TAO patients with visual defect, and examine the relative important risks in the pathogenesis of retinal perfusion alteration. It might help us understand the high risks of altered retinal perfusion and the underlying mechanism of VF defect in TAO.

## Methods

### Subjects and basic examination

All TAO patients were recruited from the Eye Hospital of Wenzhou Medical University, Wenzhou, China. The TAO diagnosis was decided by one professor, based on the criteria of Bartley (10). The patients with anti-glaucoma or antihypertension treatment, or other eye diseases (such as glaucoma, uveitis, and other retinal diseases) as well as systemic diseases (like diabetic retinopathy), were excluded. The patients who smoked were also excluded (11). All patients included were provided with informed consent. The current study was approved by the Ethics Committee Board, the Eye Hospital of Wenzhou Medical University, Wenzhou, China.

All TAO patients were given a comprehensive ophthalmic examination, including spherical equivalents with best-corrected visual acuity (BCVA) and slit-lamp examination. The VF test was done by a Humphrey field analyzer (SITA standard algorithms with a 30-2 program). All included VF results were with the reliable scans as false-positive error <15%, false-negative error <15%, and fixation loss <20%. The VF test with mean deviation (MD) ≤-2 dB was considered as a VF defect. We divided the TAO patients into two groups depending on their VF test result: (1) VF defect and (2) no VF defect. If one TAO patient has one eye with VF defect and the other without VF defect, it would be excluded. For both eyes with VF defect in the same TAO patients, only the severer eye would be included for analysis. For both eyes without VF defect in the same TAO patients, only the milder eye would be included for analysis. The orbital computed tomography (CT) scans were done to evaluate the ophthalmic rectus muscular index (MI) (12). Among all scanning images, only the image halfway between the posterior globe and the orbital apex was chosen to evaluate the ophthalmic rectus muscular index (MI) (12). The horizontal MI was defined as the percentage of orbital width occupied by the medial rectus and lateral rectus along the line through the optic nerve, while the vertical MI was defined as the percentage of orbital height occupied by the superior rectus and inferior rectus along the line through the optic nerve (12).

### Diurnal measurements of blood pressure and intraocular pressure

Diurnal measurements of blood pressure (measured by a validated automatic sphygmomanometer, Omron, Tokyo, Japan) and intraocular pressure (IOP, measured by the Full Auto Tonometer TX-F; Topcon, Tokyo, Japan) were collected at 05:00, 07:00, 10:00, 14:00, 18:00, and 22:00 in a single day. The six time points chosen for measurement provided sufficient time to observe diurnal rhythms of blood pressure and IOP. All patients were forbidden to do any physical activities (like swimming) or have any alcohol (or caffeine), which could affect blood pressure and IOP. Both blood pressure and IOP were measured after patients had sat and rested for at least 5 min. The pulse pressure (PP) was defined as the difference value between systolic and diastolic blood pressure. The mean PP and IOP were calculated by the average value among six measurement points, and the fluctuation of PP and IOP was defined as its standard deviation (SD) value among six measurement points.

### Optical coherence tomography angiography measurement

**Figure 1:**
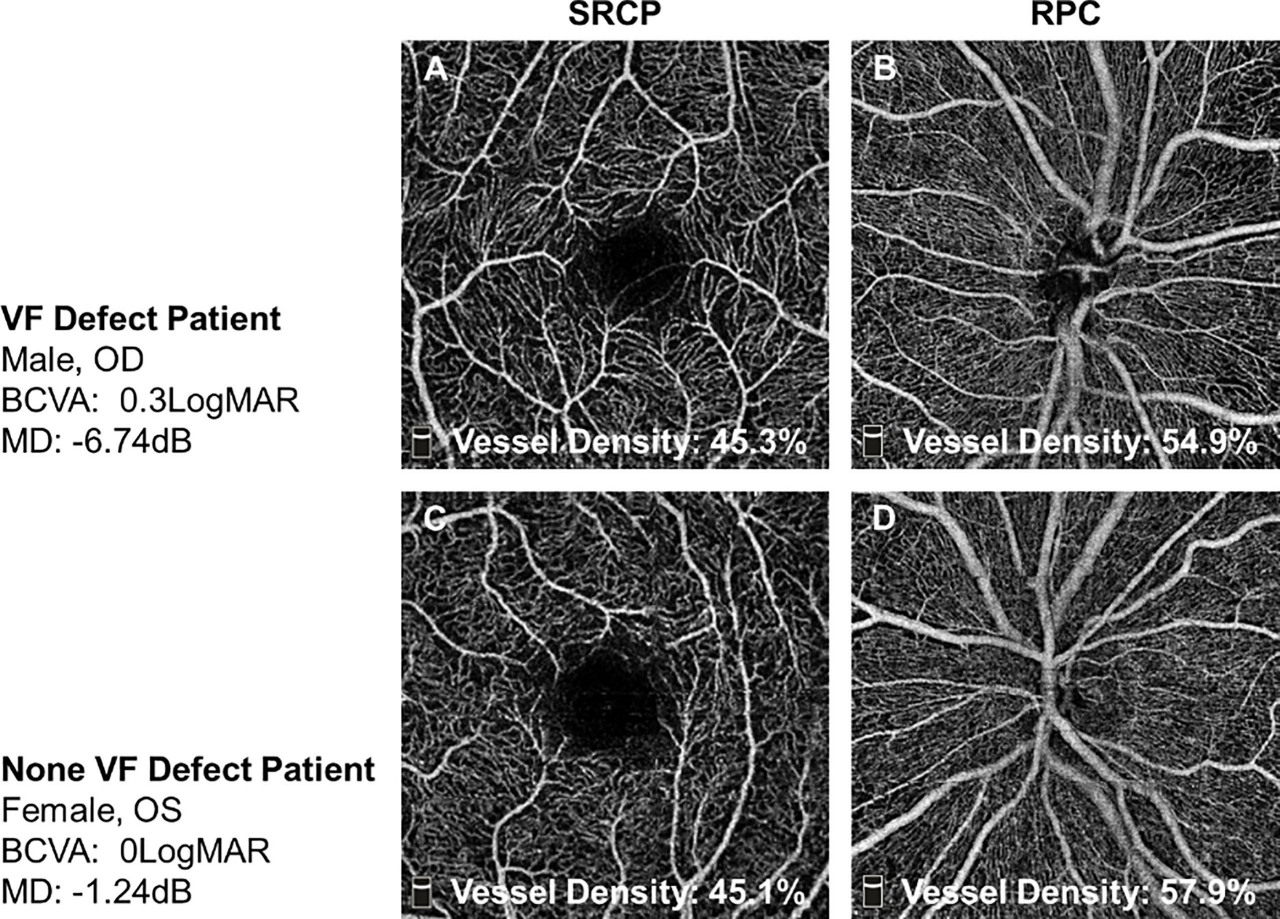
The representative OCTA images of TAO patients with or without VF defect. **(A, B)** In one VF defect TAO patient, his whole SRCP density was 45.3% and whole RPC density was 54.9%. **(C, D)** In one no-VF defect TAO patient, her whole SRCP density was 45.1% and whole RPC density was 57.9%. SRCP, superficial retinal capillary plexus; RPC, radial peripapillary capillary.

The optical coherence tomography angiography (OCTA) system (AngioVue; Optovue, Fremont, CA, USA), with a scanning speed of 70,000 A-scans per second, was used to image the retinal capillary distribution. The radial peripapillary capillary (RPC) imaging was centered on the optic nerve head with a scan size of 4.5 × 4.5 mm, and the superficial retinal capillary plexus (SRCP) imaging was centered on the fovea with the scan size of 3.0 × 3.0 mm (**Figure 1**). The RPC layer was extended from the internal limiting membrane (ILM) to the nerve fiber layer (NFL), and the SRCP layer was extended from ILM to 10 μm above the inner plexiform layer (IPL), which were segmented by a built-in program. From the report of the built-in program, we could get the vessel density of RPC and SRCP. The RPC density was defined as the percentage of small vascular area in the whole analyzed area. The whole RPC-analyzed area was a ring with a 2-mm inner diameter and a 4-mm outer diameter centered on the optic disc which was further separated into two sectors (superior and inferior sectors). The SRCP density was defined as the percentage of vascular area in the whole analyzed area. The whole SRCP-analyzed area was a ring with a 1-mm inner diameter and a 3-mm outer diameter centered on the fovea which was further separated into another four sectors (superior, nasal, inferior, and temporal sectors). The peripapillary retinal nerve fiber layer (pRNFL) thickness was from the same report of the built-in program as RPC density, which was separated into two areas (superior and inferior sectors) as well. The OCTA images with obvious eye movement, quality less than 4/10 (defined by the machine itself), wrong layer segmentation, etc., would be excluded. One masked reader checked all the OCTA images.

### Statistical analysis

All continuous data were shown in the form of means ± SD and analyzed by SPSS 22.0 (SPSS, Inc., Chicago, IL, USA). The spherical equivalent (SE) was calculated as the spherical power plus one-half of the cylindrical power, and BCVA was converted into the form of the logarithm of minimal angle resolution (LogMAR). The independent t-test was used to compare the parameters between two groups, and χ ^2^ was used to test the gender difference. Pearson’s correlation and general estimating equation (GEE) were used to calculate relationships among retinal capillary density, age, IOP, MI, and other related parameters. The GEE was also used to adjust the IOP influence on the RPC density, SRCP density, and pRNFL thickness between the two groups. The P-value less than 0.05 was considered to be statistically significant.

## Results

### Basic information

Sixty-two TAO patients (39 with VF defect and 23 without VF defect) were included. The MD was -7.39 ± 6.36 dB in TAO with VF defect and -0.54 ± 0.79 dB in TAO without VF defect (P < 0.001). There was no significant difference in age, sex, and SE (P = 0.648, 0.149, and 0.109, **Table 1**) between the two groups. The TAO patients with VF defect were with worse BCVA and larger horizontal and vertical MI compared to the TAO patients without VF defect (P = 0.007, 0.006, and <0.001, **Table 1**).

**Table 1:** Basic information of all TAO patients.

	VF defect	None VF defect	P
N	39	23	–
Age, year	55 ± 8	54 ± 7	0.648
Gender, M: F	24:15	9:14	0.149
SE, diopter	-0.04 ± 1.63	-0.76 ± 1.70	0.109
BCVA, LogMAR	0.23 ± 0.24	0.09 ± 0.14	0.007
MD, dB	-7.39 ± 6.36	-0.54 ± 0.79	< 0.001
Horizontal MI	0.55 ± 0.11	0.46 ± 0.12	0.006
Vertical MI	0.64 ± 0.12	0.51 ± 0.11	< 0.001

M, male; F, female; SE, spherical equivalent; BCVA, best-corrected visual acuity; MD, mean deviation; MI, muscle index; VF, visual field.

### Diurnal rhythm of pulse pressure and IOP

**Figure 2:**
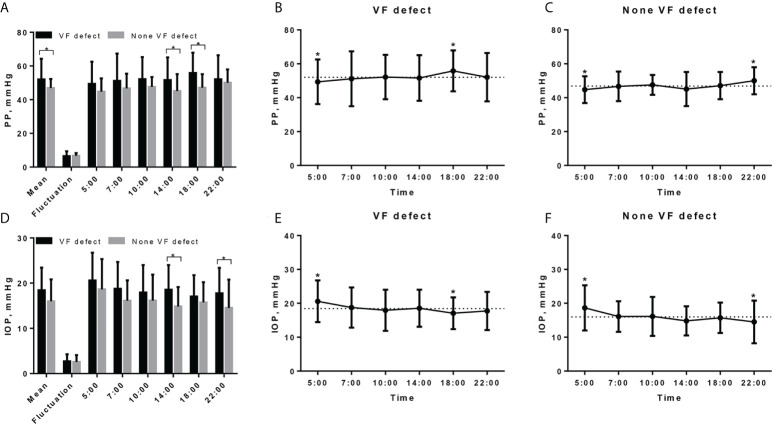
Diurnal rhythm of pulse pressure and IOP in TAO patients with and without VF defect. **(A)** Diurnal rhythm of PP in TAO patients with and without VF defect, * indicated significant difference between two groups. **(B)** Diurnal rhythm of PP in TAO patients with VF defect, dashed line indicates the mean PP value (52.06 mmHg) and * indicates the significant difference between PP value at that measurement point and mean value. **(C)** Diurnal rhythm of PP in TAO patients without VF defect, dashed line indicates the mean PP value (46.87 mmHg) and * indicates the significant difference between PP value at that measurement point and mean value. **(D)** Diurnal rhythm of IOP in TAO patients with and without VF defect, * indicates significant difference between two groups. **(E)** Diurnal rhythm of IOP in TAO patients with VF defect, dashed line indicates the mean IOP value (18.44 mmHg) and * indicates the significant difference between IOP value at that measurement point and mean value. **(F)** Diurnal rhythm of IOP in TAO patients without VF defect; dashed line indicates the mean PP value (15.99 mmHg) and * indicates the significant difference between PP value at that measurement point and mean value. PP, pulse pressure; IOP, intraocular pressure; VF, visual field.

The mean PP was higher in TAO patients with VF defect when compared to the patients without VF defect (52.06 ± 12.27 vs. 46.87 ± 5.40 mmHg, P = 0.026, **Table 2**; **Figure 2A**), although the significant difference of PP between the two groups was found only at 14:00 and 18:00 (**Figure 2A**). Both TAO patients with and without VF defect had the lowest PP at 5:00, while TAO patients with VF defect had the highest PP at 18:00 and patients without VF defect had the highest PP at 22:00 (**Figures 2B, C**). For the PP fluctuation, there was no difference in TAO patients with and without VF defect (P = 0.733, **Table 2**, **Figure 2A**).

**Table 2:** Blood pressure and IOP information in all TAO patients.

	VF defect	None VF defect	P
PP, mmHg			
Mean	52.06 ± 12.27	46.87 ± 5.40	0.026
Fluctuation	6.49 ± 2.91	6.71 ± 1.56	0.733
IOP, mmHg			
Mean	18.44 ± 5.00	15.99 ± 4.85	0.065
Fluctuation	2.72 ± 1.55	2.57 ± 1.53	0.700

PP, pulse pressure; IOP, intraocular pressure; VF, visual field.

Although the mean IOP was higher in TAO patients with VF defect than in patients without VF defect, there was no significant statistical difference (18.44 ± 5.00 vs. 15.99 ± 4.85 mmHg, P = 0.065, **Table 2**; **Figure 2D**). Different from the diurnal rhythm of PP, both TAO patients with and without VF defect had the highest IOP at 5:00, while TAO patients with VF defect had the lowest IOP at 18:00 and patients without VF defect had the lowest IOP at 22:00 (**Figures 2E, 2F**). The IOP fluctuation did not differ between TAO patients with and without VF defect (P = 0.700, **Table 2**; **Figure 2D**).

### Alteration of retinal capillary density, pRNFL thickness, and its associations with each other and with VF defect

The representative OCTA images from the two groups are shown in **Figure 1**. The whole RPC density was significantly decreased in TAO patients with VF defect when compared to the patients without VF defect (51.86 ± 3.43 vs. 53.51 ± 2.58, P = 0.025, **Table 3**). The same alteration tendency of RPC density was found in the superior peripapillary area (P = 0.015, **Table 3**) but not inferior peripapillary areas (P = 0.078, **Table 3**). Not only the whole SRCP density but also SRCP density in five separated areas were not significantly different between the TAO patients with and without VF defect (P = 0.143~0.436, **Table 3**). For the pRNFL thickness, neither the whole pRNFL thickness nor the pRNFL thickness in two respective areas did not show any significant difference between the TAO patients with VF defect and without VF defect (P = 0.056~0.113, **Table 3**).

**Table 3:** Alteration of retinal capillary density and peripapillary RNFL thickness.

	VF defect	None VF defect	P
RPC density, %			
Whole	51.86 ± 3.43	53.51 ± 2.58	0.025 (0.021)
Superior	51.53 ± 4.14	53.71 ± 2.86	0.015 (0.008)
Inferior	52.19 ± 3.12	53.31 ± 2.69	0.078 (0.052)
SRCP density, %			
Whole	41.09 ± 4.72	41.73 ± 5.63	0.314 (0.459)
Superior	45.59 ± 6.52	47.39 ± 6.16	0.143 (0.225)
Nasal	44.05 ± 4.67	44.30 ± 6.92	0.432 (0.436)
Inferior	45.56 ± 5.06	46.52 ± 6.24	0.256 (0.450)
Temporal	43.39 ± 5.90	43.09 ± 8.54	0.436 (0.318)
pRNFL thickness, μm			
Whole	113.79 ± 14.32	118.26± 7.50	0.056 (0.055)
Superior	114.36 ± 16.35	118.39 ± 9.70	0.113 (0.057)
Inferior	113.44 ± 15.02	118.00 ± 7.71	0.060 (0.051)

M, male; F, female; SE, spherical equivalent; BCVA, best-corrected visual acuity; MD, mean deviation; MI, muscle index; VF, visual field. P values in parentheses were the P values after the adjustment for the IOP by GEE analysis.

When we further did Pearson’s correlation of RPC/SRCP density with pRNFL thickness, we only included their corresponding data in the whole analyzed area. There was a significant correlation between RPC density and pRNFL thickness (r = 0.314, P = 0.013), while no correlation was found between SRCP density and pRNFL thickness (r = 0.106, P = 0.412).

When we further did Pearson’s correlation of RPC and SRCP with VF MD value, we only included their density in the whole analyzed area. There was a significant correlation between RPC density and VF MD value (r = 0.242, P = 0.029), while no correlation was found between SRCP density and VF MD value (r = 0.026, P = 0.419).

### Influence on RPC density

In the univariable GEE analysis with the RPC density as the outcome factor, the mean PP and its fluctuation (P = 0.013 and 0.035, respectively) were statistically significant, while age, gender, SE, vertical MI, horizontal MI, mean IOP, and its fluctuation were not significant (P = 0.067~0.860, **Table 4**). Only the parameters with P < 0.05 in univariable GEE analysis were included for further multivariable analysis. In the final multivariable GEE analysis (**Table 4**), the increased RPC density was only associated with less PP (β = -0.082, standard error = 0.038, P = 0.029), but not associated with PP fluctuation (P = 0.080).

**Table 4:** The GEE analysis with RPC as the predictor factor.

Parameters	Univariable			Multivariable		
	β	Standard error	P	β	Standard error	P
Age, year	-0.075	0.055	0.172	–	–	–
Gender, Male	1.225	0.817	0.134	–	–	–
SE, diopter	-0.044	0.249	0.860	–	–	–
Horizontal MI	-6.096	3.333	0.067	–	–	–
Vertical MI	-3.377	3.050	0.268	–	–	–
PP mean	-0.094	0.038	0.013	-0.082	0.038	0.029
PP Fluctuation	-0.343	0.163	0.035	-0.279	0.159	0.080
IOP mean	-0.063	0.083	0.449	–	–	–
IOP Fluctuation	0.197	0.271	0.467	–	–	–

SE, spherical equivalent; MI, muscle index; PP, pulse pressure; IOP, intraocular pressure; GEE, general estimating equation.

## Discussion

In the current study, we used OCTA to evaluate retinal vessel alteration in TAO patients. Our findings were consistent with most previous papers that TAO was with reduced retinal perfusion (**Table 5**). However, few of those papers focused on its risk factors and relation with VF defect in TAO patients. From our results, we demonstrated a strong association of elevated pulse pressure with reduced RPC perfusion in TAO patients with VF defect. It might support the importance of the “system vascular” factor (as reflected by PP) rather than the “mechanical” factor (as reflected by MI) in the pathogenesis of reduced retinal perfusion in TAO patients, which would further lead to visual field defect.

**Table 5:** Summary of previous studies on retinal perfusion changes in TAO.

Study	Groups	Retinal perfusion changes
Current study	TAO (1) with and (2) without VF defect	The reduced RPC density in TAO patients with VF defect compared to patients without VF defect.
Zhang et al.^5^	TAO (1) with and (2) without DON	The reduced RPC density and unchanged SRCP density in TAO patients with DON compared to patients without DON.
Wu et al.^13^	(1) TAO with DON; (2) normal controls.	The reduced SRCP density in TAO patients with DON compared to normal controls.
Wu et al.^14^	(1) TAO; (2) normal controls.	The reduced SRCP density in TAO patients compared to normal controls.
Yang et al.^15^	(1) Severe inactive TAO; (2) normal controls.	The retinal venous diameter decreased significantly in severe TAO.
Tehrani et al.^16^	(1) Active TAO; (2) not active not compressive TAO; (3) normal controls.	The reduced RPC density and SRCP density in active TAO patients compared to not active not compressive TAO patients and normal controls.
Wu et al.^17^	TAO (1) with and (2) without DON	The reduced RPC density in TAO patients with DON compared to patients without DON.

TAO, thyroid-associated ophthalmology; RPC, radial peripapillary capillary; VF, visual field; SRCP, superficial retinal capillary plexus; DON, dysthyroid optic neuropathy.

We found the decreased RPC perfusion in TAO patients with VF defect but not a significant alteration of SRCP perfusion when compared to the TAO patients without VF defect in the current study. The peripapillary vessels might be impaired more easily during the process of TAO. As an optic neuropathy, peripapillary vessels were more directly influenced as they were the branches of large blood vessels that went out from the optic disc. Moreover, different from macular vessels with frequent anastomoses as a long and straight form, the structure of peripapillary vessels might lead it to be impaired more easily than macular vessels. The peripapillary vessel had higher diagnostic accuracy to differentiate TAO patients with dysthyroid optic neuropathy (DON) and no-DON (5).

The reduced peripapillary vessel density was correlated with the degree of VF defect in TAO patients. The VF defect, as the result of several optic neuropathies, had been reported to be with peripapillary degeneration (5). The peripapillary vessels were essential to supplying oxygen and nutrition to the peripapillary tissue. The degeneration of the peripapillary tissue would further lead to reduced peripapillary perfusion, vice versa, resulting in VF defect.

How and why some TAO eyes had reduced retinal perfusion and resulted in VF defect, while others did not, were still unclear and likely involved multiple factors. In the current study, we considered MI and PP as biomarkers for the “mechanical” and “system vascular” factors, respectively. The reduced retinal perfusion in TAO patients might be associated with both two factors. For the “mechanical” factor with orbital apex compression, the increased orbital volume resulted in the elevated retrobulbar space pressure with sustained stretching of the optic nerve and altered venous drainage (1, 4). However, the resistance index of the ophthalmic artery did not alter after orbital decompression in some TAO eyes (18). Less than half of TAO patients with compressive optic neuropathy had signs of optic swelling (19). Even rarely, optic nerve stretch was found from the CT in some TAO patients with extreme proptosis (3). Moreover, despite successful orbital decompression being done, some TAO patients still complained about progressively decreased visual function (2). Based on these phenomena, we hypothesized that besides the “mechanical” theory, there might be other important aspects of mechanisms associated with visual impairment in TAO patients.

From our multivariable GEE analysis results, the abnormal PP was more associated with the peripapillary perfusion compared to the “mechanical” factor of the enlarged muscle in TAO patients. The abnormal blood pressure would play a more important role in the retinal vessel changes even previous to the mechanical compression (20). In TAO patients with VF defect, they showed higher PP than patients without VF defect and it was correlated with the decreased retinal perfusion. The decreasing systemic vascular resistance in TAO may lead to increased systolic pressure and relatively reduced diastolic pressure, resulting in elevated PP (5, 21–23). The TAO patients were always with arterial stiffness, which led to the breakdown of blood pressure autoregulation and the expansion of pulse pressure (24). The continually abnormal blood pressure then contributed to end-organ damage, such as impairment of retinal vessels (6, 7). The abnormal deposition of the antibodies in the vasculature was related to the inflammatory process, which further led to the alteration of capillary density in TAO eyes (25–27). Due to the impact of arterial stenosis and venous stasis, retinal vessel perfusion was hindered (18, 28–30). The abnormal elevated PP during the progression of TAO with VF defect might be a sign of a vascular anomaly. At the same time, the elevated PP impaired the choroidal circulation and oxygen supplement, which further influenced the retinal perfusion (31). Moreover, the elevated PP also led to the decreased pressure of the cerebrospinal fluid and higher trans-lamina cribrosa pressure, resulting in the degeneration of the optic nerve (32–34). All the above might explain the association between the abnormal PP and RPC in TAO with VF defect. Altered PP would be a high risk for retinal perfusion damage with a VF defect in TAO.

There were still some limitations in our current study. The sample size we included was small, which still should be enlarged to further confirm our findings. We only measured the diurnal rhythms of the blood pressure and IOP but not the OCTA parameters in the current study. With the data of diurnal rhythms for OCTA parameters, the current conclusion of our study would be stronger, although it had been reported that these OCTA parameters would not be variable (34). We would also like to do a follow-up with all TAO patients to further confirm that abnormal PP might be a risk of the reduced peripapillary perfusion with a VF defect.

In conclusion, we demonstrated that PP but not MI was more associated with reduced RPC in TAO, which further led to visual field defect. The elevated PP was a high risk of reduced retinal peripapillary perfusion and visual field defect, although the interrelationships among the blood pressure, retinal perfusion, and visual function in TAO patients still warrant further study.

## Data availability statement

The raw data supporting the conclusions of this article will be made available by the authors, without undue reservation.

## Ethics statement

This study was reviewed and approved by the Ethics Committee Board, the Eye Hospital of Wenzhou Medical University, Wenzhou, China. The patients/participants provided their written informed consent to participate in this study.

## Author contributions

JY, WW and YT contributed to the conception and design of the study. JY, WL and XH determined the experimental methods. JY, HXJ, MX and HCJ performed the experiments. JY, MW, ZL and QC analyzed and interpreted the data. JY and YT wrote and modified the manuscript. All authors contributed to the article and approved the submitted version.

## Funding

This study is supported by research grants from the Medical Health Science and Technology Project of Zhejiang Provincial Health Commission (Grant No. 2020KY192).

## Conflict of interest

The authors declare that the research was conducted in the absence of any commercial or financial relationships that could be construed as a potential conflict of interest.

## Publisher’s note

All claims expressed in this article are solely those of the authors and do not necessarily represent those of their affiliated organizations, or those of the publisher, the editors and the reviewers. Any product that may be evaluated in this article, or claim that may be made by its manufacturer, is not guaranteed or endorsed by the publisher.
